# Another Case of Maggots in the Nasal Cavity

**Published:** 1887-08

**Authors:** F. A. Schmitt

**Affiliations:** Schulenberg, Texas


### ANOTHER CASE OF LIVE MAGGOTS WITHIN THE NASAL CAVITY AND PHARYNX.

*By F. A. Schmitt, M. D., Schulenberg, Texas.*

For Daniel's Texas Medical Journal.

ON FRIDAY, the 17th of June, I was called to A. B., male, aged 18, suffering, as I was told, from severe neuralgic pains of left side of face. I found patient in a comatose condition, pulse 140, temperature 104, oozing of bloody serum from nose. On inspection of throat, after forcibly opening his mouth, observed tonsils and uvula œdematous and imflamed; soft palate, bulged out, covering last molar teeth. Had vomited up *two live* maggots just before my arrival. This, and my experience in reported former case, satisfied me as to nature of case, and immediately initiated treatment accordingly.

A female catheter, containing a quantity of calomel, perhaps one drachm—I inserted into the nostrils to near the posterior nares, and, forcibly blowing into the tube from without, I endeavored to scatter the drug as near the worms as possible. The same was done by placing the instrument through the mouth behind the soft palate. After the lapse of perhaps one hour, in a fit of coughing and sneezing, three worms were expelled, a few minutes afterwards twenty, and within two hours after instituting treatment one hundred and eighty seven maggots had been made sick enough to leave their hiding place.

The boy had now regained consciousness, and I advised a strong solution (mixture) of quinine and water to be injected into the nasal cavities every hour. On calling the next day, eighty-four more worms had been expelled from mouth and nose, several had been found in the bedding, and quite a number in a watery evacuation from the bowels, which he had had a few hours after my departure—probably in consequence of the action of absorbed and swallowed calomel—so that, in all, over three hundred worms had lodged within him, being nearly the number (350) which had been expelled in my former reported case. Nine hours—from 2 p. m. to to 11 p. m., were required to displace them.

The worms, in this case, having seemingly had but time to prey on superficial structures (mucous membrane), leaving the patient to suffer but little afterwards. He is now convalescent, and in a few days will be restored to perfect health.

